# Evaluation of left ventricular remodelling in young Afro-Caribbean athletes

**DOI:** 10.1186/s12947-019-0169-8

**Published:** 2019-10-20

**Authors:** Giorgio Galanti, Loira Toncelli, Benedetta Tosi, Melissa Orlandi, Chiara Giannelli, Laura Stefani, Gabriele Mascherini, Pietro A. Modesti

**Affiliations:** Department of Experimental and Clinical Medicine-Sports Medicine and Exercise Unit, University of Florence, AOUC, Careggi, Florence, Italy

**Keywords:** Afro-Caribbean athletes (AA), Strain, Race

## Abstract

**Background:**

Cardiac adaptation to intense physical training is determined by many factors including age, gender, body size, load training and ethnicity. Despite the wide availability of ECG analysis, with a higher presence of abnormalities in different races, echocardiographic studies on young Afro-Caribean (AA) and Caucasian athletes (CA) are lacking in literature. We aimed to assess the effect in the secondary LV remodelling of load training in young AA players compared to matched CA players.

**Method:**

Seventy-seven AA and 53 CA matched soccer players (mean age 17.35 ± 0.50 and 18.25 ± 0.77 y) were enrolled. They were evaluated with echocardiography. A subgroup of 30 AA and 27 CA were followed up for a period of 4 years. The myocardial contractile function was evaluated by speckle-tracking echocardiographic global longitudinal strain (GLS).

**Results:**

No significant differences were found in weight and height and in blood pressure response to maximal ergometer test in either group. In AA a higher level of LV remodelling, consisting in higher LV wall thickness, higher interventricular septum (IVS) and posterior wall (PW) thickness were found (IVS: 10.04 ± 0.14 and 9.35 ± 0.10 in AA and CA respectively, *p* < 0.001. PW: 9.70 ± 0.20 and 9.19 ± 0.10 mm in AA and CA respectively, *p* < 0.05). Strain data showed no significant differences between the two groups (22.35 ± 0.48 and 23.38 ± 0.69 in AA (*n* = 27) and CA (*n* = 25), respectively). At the beginning of the follow-up study AA showed a significantly higher left ventricular remodelling (IVS = 9.29 ± 0.3 and 8.53 ± 0.12 mm in AA and CA respectively, *p* < 0.002. PW = 9.01 ± 0.2 and 8.40 ± 0.20 in AA and CA respectively, *p* = 0.1). During the next four years of follow-up we observed a regular parallel increase in LV wall thickness and chamber diameters in both groups, proportionally to the increase in body size and LV mass. (IVS = 10.52 ± 0.17 and 9.03 ± 0.22 mm in AA and CA respectively, *p* < 0.001. PW: 10.06 ± 0.17 and 8.26 ± 0.19 mm in AA and CA respectively, *p* < 0.001).

**Conclusion:**

The study shows that the ventricular remodelling observed in AA appears to be a specific phenotype already present in pre-adolescence. These data also suggest that genetic/ethnic factors play a central role in left ventricular remodelling during the first years of life in elite athletes.

## Introduction

Regular and intense physical exercise induces several morphological and functional heart modifications, characterizing the so-called “athlete’s heart”. This physiological “adaptive” myocardial hypertrophy is related to the intensity and kind of sport practiced. This ventricular adaptive remodelling is reversible and characterized by preserved systolic and diastolic function, resulting in a more efficient cardiac pump [1, 2]. It has been widely demonstrated that cardiovascular adaptation to sports training is influenced by many factors including body size, age, gender, life style, load training and ethnicity [3, 4]. Ethnicity is considered a determining factor in the electrical, structural and functional remodelling of the athlete’s heart [4], although knowledge regarding cardiac adaptation to exercise largely derives from echocardiographic evaluation of adult CAs. The exponential increase in the number of multiethnic athletes competing at high level highlights the lack of studies on the role of ethnicity in myocardial adaptation to exercise and the need to define ethnicity-specific patterns. It is well established that ECGs recorded in elite athletes usually present characteristic changes reflecting structural and electrical remodelling of the heart [5]. However, these abnormalities of athletes’ ECGs may be difficult to differentiate from disease which carries a risk of exercise-related sudden cardiac death. The most common training-related ECG changes [6] are sinus bradycardia, first-degree AV block, incomplete right bundle branch block, early repolarization and isolated QRS voltage criteria for left ventricular hypertrophy [7]. “Uncommon” ECG changes have also been described in literature: these uncommon patterns, mostly derived from CA ECG studies, appear to be more frequent if we consider young AC athletes [8], in whom the application of current ECG criteria would result in an abnormal ECG. In fact T-wave inversion in 2 or more contiguous leads, ST-segment depression elevation and Brugada-like repolarisation are some of the most frequently found changes in healthy AA athletes [4].

Morphological and structural myocardial adaptations to exercise have been largely studied by echocardiography. The secondary LV remodelling to load training consists of an increased LV wall thickness and an augmented LV cavity dimension with preserved systolic and diastolic function [9]. Although the normal upper limits of the athlete’s heart have been established [9], there is still a grey zone where physiological heart adaptation overlaps with cardiac diseases implicated in exercise-related sudden cardiac death [10]. This is especially true in young AA athletes whose ECG and echocardiographic patterns could be markedly altered. Despite the wide availability of ECG and echocardiographic analysis in CA, less has been done in young elite AA athletes. More data are required to detect ethnic-specific cardiac adaptations to exercise.

## Aim of the study

The aim was to study cardiovascular and, specifically, left ventricular remodelling in young African Americans compared to young Caucasian soccer players. All the athletes belonged to the same team, trained with the same load and frequency, and had the same lifestyle. The role of a specific kind of sport on cardiovascular remodelling (soccer) was studied. Furthermore, Strain analysis, not commonly calculated in adolescent athletes, was used to evaluate myocardial contractile function in ultrasound imaging.

Notably, a subgroup of the athletes enrolled in our study was evaluated for a 4-year follow-up, in order to assess how left ventricular remodelling changed in time.

## Materials and methods

### Subjects

Our study enrolled 77 young sexually developed soccer players of Afro-Caribbean origin (Afro-Caribbean athletes: AA; mean age 17.34 ± 0.50 yrs) and 53 matched Caucasian soccer players (Caucasian athletes: CA; mean age18.35 ± 0.67 yr). In agreement with the common definition in literature, the AA (Afro–Caribbean athletes), despite the fact that not all of them came strictly from America, will be called “African-Americans”. Athletes with a previous personal or family history of cardiac or pulmonary disease, family history of premature (≤40 yr) sudden cardiac death or cardiomyopathy were excluded from the study.

The athletes were members of the same soccer team, trained with the same load 6 times a week, each training session lasting ≥2 h, and they had a similar lifestyle. 30 AA and 27 CA were evaluated with echocardiography for a 4-year follow-up (mean age at first evaluation: 12.47 ± 0.60 and 13.60 ± 0.38 for AA and CA, respectively). Players who gave up football or changed team, as normally occurs in seasonal recruitment, were not included in the follow-up study (47 AA and 26 CA).

All athletes were evaluated yearly at the beginning of the season at the Sports Medicine Department of the University of Florence – Italy. They underwent assessment with a health questionnaire (to exclude any familiarity for chronic or metabolic diseases, use of illegal substances and any symptoms); physical examination, rest ECG, maximal ergometric test and 2-dimensional echocardiography as part of a pre-participation cardiac evaluation. We also evaluated contractile function using speckle-tracking echocardiographic global longitudinal strain (GLS).

All athletes provided written consent for the evaluation. Approval for the whole procedure was obtained from the Human Research and Ethics Committee of the University of Florence. The studies conformed to the Declaration of Helsinki.

### Clinical evaluation and anthropometric parameters

Athletes underwent clinical evaluation, including weight, height and blood pressure measurement at rest. Body mass index (BMI) was calculated by the formula “weight [Kg]/height [m^2^]”. Body surface area (BSA in [m^2^]) was obtained using the formula of DuBois and DuBois for BSA (0.007184 x (Height (cm) 0.725 x Weight (kg) 0.425) [11].

### ECG

Standard 12-lead ECG was performed with the subject supine, after a few minutes of rest with normal breathing, and recorded at 25 mm/s. ECG analysis was performed according to the previously described criteria [12]. Specifically, we measured heart rate (beats/min), PR interval (ms), QRS duration (ms), QT interval corrected for the heart rate (s) [13], presence of Q waves (≥2 mm in depth in ≥2 leads), R/S-wave amplitude in precordial leads (S1 + R5) (mm), and Sokolow-Lyon criterion for LV hypertrophy (positive if ≥35 mm) [14], presence and shape (concave or domed) of ST-segment elevation (≥1 mm, in ≥2 contiguous leads), presence of J-wave (≥1 mm), or ST-segment slurring, T-wave inversion (≥2 mm in depth in ≥2 contiguous leads, with exclusion of III and a VR), and flat/biphasic T-wave pattern (in ≥2 contiguous leads).

### Exercise stress testing

An upright treadmill stress test was performed with the modified Bruce protocol [15]. ECGs were recorded at 1-min intervals and blood pressure was measured in pre-test, at the apex of the exercise and at the 4th minute of recovery. Athletes exercised to volitional exhaustion and were assessed specifically for the development of abnormal or flat blood pressure response, ischemic changes, arrhythmias or symptoms [16].

### Echocardiographic study

Trans-thoracic echocardiography was conducted by two experienced and certified cardiologists using a commercially available ultrasound system: iE33 Philips Medical System (Bothell, WA). These specialists work together and therefore the reproducibility of data is high and the inter-observer variability low (< 5%). Furthermore, at least 5 double blind echo tests were carried out after 3 days, in order to confirm the overlap of the data obtained. According to the ASE guidelines [17], all the systo-diastolic LV parameters were calculated at rest in parasternal long-axis view: interventricular septum (IVS), posterior wall thickness (PW), left ventricle end-diastolic diameter (LVEDd), end-systolic diameter (LVESd), right ventricle diameter (RVd), aortic root diameter (AOR) and left atrium antero-posterior dimensions. Assessment of left ventricular mass (LVM [g/m^2^) was obtained according to the formula of Devereux and it was also indexed to BSA (LVMI [g/m^2^]) [18]. Considering the regularity of the athletes’ left ventricular chamber geometry, the ejection fraction (EF%) was calculated according to the formula (LVEDd - LVESd/ LVEDd), which assembles volumes to diameters.

LV Relative Wall Thickness (RWT) was determined as the ratio of wall thickness and end-diastolic diameter according to the formula: [2x(PWTd+IVSd)/LVDd] [19].

Left ventricular hypertrophy (LVH) was defined as a maximal LVMI of 102 g/m^2^, in agreement with current guidelines [19].

Right Ventricle (RV) chamber was studied morphologically from long axis view (RV diameter) and investigated functionally by apex four-chamber view (contractile function and TAPSE).

Diastolic parameters were measured by Doppler analysis in the presence of a stable RR interval and in three different but sequential measurements from the four-chamber view. Transmitral flow of E and A wave peak velocities, isovolumetric relaxation, deceleration times and E/A ratio were obtained. Eventual presence of valve insufficiency or stenosis was determined by continuous wave and colour Doppler analysis from the five-chamber view. The echocardiographic evaluation was also useful for excluding any cardiac abnormalities, i.e. interventricular or interatrial septal defects, or any pulmonary hypertension and other congenital heart disease.

The 2D images of four-chamber views were post-processed with X-Strain software (Philips Q-station) to provide an angle-independent tool for the evaluation of velocities and strain. This software allows automatic evaluation of both endocardial and sub-endocardial edges from 2D B-mode echocardiographic clips recorded at rest. Strain analysis by speckle tracking is independent of translational motion and of tethering effects of the nearby regions, allowing uniformity of measurements through the normal LV myocardium. The endocardial border is drawn by the operator in a four-chamber view on a single frame from one annulus to another; the first and last points delineate the mitral plane. Since the global longitudinal strain (GLS) has better reproducibility than other deformation indices [20], this evaluation was preferred.

### Statistical analysis

Statistical significance was considered at *P* < 0.05. Student’s t test was conducted post hoc and reported. Data are expressed as means±SD. Comparison of change scores was undertaken using T test. The correlation between posterior wall and interventricular septum thicknesses and ECG alterations was investigated with the Spearman non parametric test.

## Results

No statistically significant differences in mean age and anthropometric parameters, as well as in lifestyle and training modalities, were present in either group (77 AA and 53 CA). Mean age, height, weight, BMI, resting heart rate and basal systolic and diastolic blood pressure (SBP, DBP) were similar in both groups (Table 1).

**Table 1:** Principal characteristics of the two groups of athletes

Parameter	AA (*n* = 77)	CA (*n* = 53)	*p*
Age (years±SE)	17 ± 0,5	18 ± 0,8	0.85
Height (cm ± SE)	173 ± 1,1	174 ± 0,9	0.93
Weight (Kg ± SE)	65 ± 1,3	66 ± 1	0.72
BMI (±SE)	21,4 ± 0,2	21,8 ± 0,2	0.56
Resting HR (AAm ± SE)	66,8 ± 0,8	64,1 ± 2	0.09
SBP (mmHg±SE)	115 ± 0,6	116 ± 1,3	0.52
DBP (mmHg±SE)	73 ± 0,5	70 ± 1,5	0.04

*Legend: BMI: Body Mass Index; SBP: systolic blood pressure; DBP: diastolic blood pressure.HR:Heart Rate*

### 12 leads ECG

A basal and stress exercise 12-lead ECG was carried out with every athlete. Basal ECGs showed that altered repolarization patterns such as inverted T waves in leads V2-V4 and early ventricular repolarization were more present and statistically significant in AA compared to CA, confirming data present in literature nowadays. Specifically, 55% AA and only 7% CA showed these alterations [21].

### Echocardiography

Echocardiography showed parameters within normal limits in all athletes. Ultrasound analysis of the left ventricular remodeling showed normal and similar chamber diameters and volume values in both groups, with a preserved EF, but a statistically significant increase of IVS thickness. PW thickness was present in AA compared to CA. IVS thickness was 10.1 ± 0.1 and 9.4 ± 0.1 mm in AA and CA respectively; PW thickness was 9.7 ± 0.1 and 9.2 ± 0.1 mm in AA and CA, respectively. Calculation of indexed left ventricular mass (iLVM) was within the normal range and comparable in the two groups, as well as the LVEDd. End diastolic volume was significantly different in the two groups, despite being potentially in agreement with the different morphology of the ethnic characteristics and slightly enhanced by the acceptable inter-operator variability. The differences found did not support any clinical implications. The systolic and diastolic function was preserved and comparable in both groups. RV chambers showed normal diameter, function and morphology (Table 2).

**Table 2:** Principal echocardiographic parameters in AA and CA

Parameter	AA (*n* = 77)	CA (*n* = 53)	*P*
IVS(mm)	10,1 ± 0,1	9,4 ± 0,1	**< 0,001**
PW(mm)	9,7 ± 0,1	9,2 ± 0,1	**< 0.001**
RWT (%)	0,39 ± 0,1	0,38 ± 0,2	0,8
iLVM(g/m^2^)	99,5 ± 1,7	97,4 ± 1,5	0,09
LVEDd(mm)	50 ± 0,4	51 ± 0,6	0,06
LVESd(mm)	31 ± 0,5	32 ± 0,3	0,2
EDV (ml)	116 ± 2	124 ± 2	**< 0,005**
ESV (ml)	41 ± 2,8	41 ± 0,9	0,9
LA (mm)	33 ± 2,8	32,1 ± 3	0,2
RA (mm)	30,5 ± 1,2	29,8 ± 2,8	0,3
EF (%)	66 ± 0,4	66 ± 0,6	0,8
RVd (mm)	22 ± 0,2	23 ± 1,5	0,2
E (cm)	86,29 ± 2,1	85,24 ± 1,5	0,3
A (cm)	44,44 ± 2,1	44,78 ± 0,9	0,7
IVRT (t)	72,1 ± 1,2	73,8 ± 0,8	0,7
DT (cm)	188,9 ± 11,9	172,9 ± 2,5	0,07
E/A	1,96 ± 1	1,94 ± 1,2	0,08

*Legend. PW: Posterior Wall; IVS: Inter Ventricular Septum; RWT: relative wall thickness; iLVM*: *Left Ventricular Mass index. LVEDV: Left Ventricle End Diastolic Volume; LVESV: Left Ventricle End Systolic Volume; ESV: End Systolic Volume; LA: left atrium; RA: right atrium; EF: Ejection Fraction; RVd: Right Ventricle diameter; E wave; A wave; IVTR: isovolumic relaxation time; DT: Deceleration Time; A statistically significant difference in IVS thickness is present in AA compared to* CA.

Data in bold are significant

In the AA group we also looked for the possible presence of a correlation between the alterations of ventricular repolarization observed in baseline ECG and the values of wall thickness with the Spearman non-parametric test. The analysis showed no significant correlation between ECG changes and the thickening degree of the IVS and PW.

### Global longitudinal strain

Strain analysis was performed in a small and apparently homogeneous subgroup of players, in terms of age, training and anthropometrical parameters. It was calculated in order to to have a deeper evaluation of the myocardial contractile function of the subjects enrolled.

The group included only 27 AA and 25 CA (mean age 18 yrs. for both groups) in order to find praecox impairment in systolic function. Results of GLS were within normal limits in both groups, without any significant difference: − 22.35 ± 0.48 and − 23.38 ± 0.69 in AA and CA.

### 5. Follow-up period

Thirty AA and 27 CA were selected from the main group and were followed-up annually for 4 years with the same clinical evaluation. At the first evaluation performed in our study, mean age, height, weight, BMI, resting heart rate and basal systolic and diastolic blood pressure were similar in both groups (Table 3).

**Table 3:** Principal characteristics of the athletes at first evaluation and after the 4-year follow-up

	First Evaluation			4-year follow-up		
	AA (*n* = 30)	CA (*n* = 27)	*p*	AA(*n* = 30)	CA (*n* = 27)	*p*
Age (years±SE)	12,5 ± 0,6	13,9 ± 0,5	0,06			
Height (cm ± SE)	162 ± 9	167 ± 3	0,08	176,4 ± 2,6	172,9 ± 2	*0,09*
Weight (Kg ± SE)	57 ± 4,4	56 ± 3,1	0,2	69,2 ± 2,8	63,5 ± 2,2	0,2
BMI (kg/m^2^ ± SE)	19,3 ± 1,2	19,8 ± 0,5	0,3	22,6 ± 0,4	21,1 ± 0,3	< 0,05
Resting HR (AAm ± SE)	63,9 ± 3,7	66,6 ± 2,9	0,09	67,5 ± 2,2	61,5 ± 3,1	0,07
SBP (mmHg±SE)	110 ± 6	116 ± 2	0,07	116,6 ± 1,4	114,7 ± 6,1	0,09
DBP (mmHg±SE)	69 ± 4	71 ± 2	0,07	73,8 ± 1,6	69,1 ± 4,1	0,08

*Legend. BMI: Body Mass Index; SBP: Systolic Blood Pressure; DBP: Diastolic Blood Pressure* After 4 years, mean age, height, weight, BMI, resting heart rate and basal systo-diastolic blood pressure continued without any statistically significant difference between the two groups

Thirty AA and 27 CA were selected from the main group and were evaluated annually in a 4-year follow-up at the Sports Medicine Department of the University of Florence - Italy. The evaluation, which allowed for follow-up of the athletes during peripuberal development, consisted in a clinical examination, basal and stress exercise ECG and echocardiography. At the first evaluation (mean age around 12–13 yrs. in both groups), statistically significant differences in left ventricular remodeling were observed between the two groups. Despite the heart chambers diameters were comparable, IVS thickness significantly increased in AA compared to CA, with a trend to an increase in PW thickness and also in iLVM**.** Our data showed that a significant increase in wall thickness appears to be present already in prepuberal age.

At first evaluation the statistically significant difference in IVS thickness and the trend in increase of PW thickness and iLVM in AA compared to CA can be noted. During follow-up the statistically significant difference in IVS and PW thickness and the trend in increase of iLVM in AA compared to CA can be noted.

At the 4-year follow-up evaluation, a comparable increase in echocardiographic parameters was observed in both groups. IVS and PW thickness and iLVM significantly increased in AA compared to CA, whereas EDd values were within normal range and showed no significant difference between the two groups. LVMI showed a mild increase in AA at the 4-year evaluation, but all the other parameters evaluated were within normal ranges and no contractile impairment was detected (Table 4).

**Table 4:** Values of parietal/wall thickness and chamber volumes in both groups at first evaluation and after 4 years

	First evaluation			4-year follow-up		
	AA (*n* = 30)	CA (*n* = 27)	*p*	AA (*n* = 30)	CA (*n* = 27)	*p*
IVS(mm)	9,3 ± 0,3	8,5 ± 0,1	< 0,02	10,5 ± 0,2	9 ± 0,2	< 0,001
PW(mm)	9 ± 0,2	8,4 ± 0,2	0,1	10,1 ± 0,2	8,3 ± 0,2	< 0,001
RWT (%)	38,2 ± 0,9	38 ± 0,5	0,8	39,4 ± 0,2	39,1 ± 0,6	0,7
iLVM(g/m^2^)	96,7 ± 5,4	90,2 ± 3,3	0,1	110,1 ± 2,3	100,5 ± 4,4	< 0,05
LVEDd(mm)	46,5 ± 1,8	48,4 ± 1,1	0,5	50 ± 0,5	50 ± 1	0,7
LVESd(mm)	29,8 ± 0,8	30,4 ± 0,9	0,6	32 ± 0,6	31 ± 1	0,6
EDV (ml)	107,1 ± 7	110,8 ± 6	0,09	120 ± 4	117 ± 4	0,5
ESV (ml)	33,6 ± 2,8	37,1 ± 2,7	0,07	41 ± 2	39 ± 2	0,09
LA (mm)	35 ± 1,6	34,2 ± 1	0,8	37,8 ± 0,8	36,4 ± 0,4	0,6
RA (mm)	33,8 ± 3,5	34,2 ± 1,7	0,6	34,5 ± 2,6	34,1 ± 2,6	0,6
EF (%)	65,2 ± 1	66 ± 1	0,3	66,5 ± 1,2	65,1 ± 2,9	0,5
RVd(mm)	21,7 ± 0,6	19,4 ± 0,5	0,006	23,6 ± 0,6	21,4 ± 0,7	0,02
E (cm)	91,4 ± 6,4	90 ± 3,1	0,3	81,4 ± 3,4	82,6 ± 3,4	0,2
A (cm)	46,6 ± 3,4	47,7 ± 3,7	0,4	45,9 ± 3,3	42,8 ± 2,6	0,08
IVRT (t)	70,6 ± 4,8	74,4 ± 3	0,09	74,4 ± 2,8	76,1 ± 2,1	0,4
DT (cm)	164,4 ± 14,6	165 ± 6	0,5	179,3 ± 11,2	176,4 ± 6,2	0,07
E/A	1,9 ± 3,5	1,9 ± 1	0,9	1,8 ± 1,1	2 ± 1,2	0,3

*Legend. PW: Posterior Wall; IVS: Inter Ventricular Septum; RWT: relative wall thickness; iLVM index; LVEDV: Left Ventricle End Diastolic Volume; LVESV: Left Ventricle End Systolic Volume; ESV: End Systolic Volume; LA: left atrium; RA: right atrium; EF: Ejection Fraction; RVd: Right Ventricle diameter; E wave; A wave; IVTR: isovolumic relaxation time; DT: Deceleration Time*

## Discussion and conclusions

The results of the study show that cardiovascular and, specifically, left ventricular remodelling is different in African Americans and Caucasian athletes and this difference is already present at an early age.

In fact AA show a higher frequency of ECG repolarization changes, particularly early repolarization patterns and inversed T waves, compared to CA. Since ethnicity was the principal differential characteristic between the groups, the presence of these ECG alterations could be attributed to genetic/ethnic factors. The results of our study confirm the data present in literature: early repolarization pattern, which is characteristic of athlete’s heart, has a higher frequency in African American athletes compared to Caucasian athletes in all the publications (60% vs 30% in adult athletes, 11% vs 1% in adolescent athletes, in African American and Caucasian athletes, respectively). These alterations are more frequent in African American sedentary individuals as well, suggesting a correlation with genetic factors rather than with exercise [21].

Data in literature also confirm the higher frequency of inversed T waves in precordial leads, which were found in 55% of AA and 7% of CA in our study. A 13% higher frequency of inversed T waves was described in African American athletes compared to Caucasian athletes, with a prevalence that varied between 12,7 and 23% [21]. The prevalence of inverted T waves in our study was higher (55%), possibly due to the fact that all the individuals enrolled were elite athletes. The higher prevalence of inverted T waves in individuals of African American descent could be caused by two mechanisms: ethnical and exercise-correlated. Data in literature, in fact, show that 10,1% of sedentary African Americans present this pattern but with a lower frequency compared to African American athletes. Inversed T waves extend until V3-V4 in African American athletes, while Caucasian athletes show this pattern only in V1-V2 [22].

Early repolarization pattern and inversed T waves were only present in inferior precordial but not in inferolateral leads (V5-V6), according to literature [22]. This permitted excluding HCM and ARVD, where inverted T waves are present in 70% of the patients and extend to inferolateral leads [23].

Our results also confirm data from Sheikh [21], who studied adolescent African American athletes and found a higher prevalence of early repolarization (91% vs 56%), left ventricular hypertrophy (89% vs 42%), and deep inverted T waves (14% vs 3%) compared to Caucasian athletes.

Echo data show significant differences in AA wall thickness (WT) compared to CA and that this pattern already appears in pre-pubertal age, (12–13 year-olds). Higher WT is also maintained after a 4-year follow-up. Besides, our study shows that left ventricular remodelling in African American athletes is a specific phenotype already present in adolescence, suggesting the role of ethnic and genetic factors in its development. Other differential elements (i.e. sex, age, kind of training) were not present.

The results of our study confirm data in literature: many publications report left ventricular WT 1–2 mm higher in African American athletes compared to Caucasians (i.e. 11.3 ± 1.6 vs 10.0 ± 1.5 mm), with 13% of African American athletes having a WT > 12 mm and 1,1% > 16 mm, in the absence of HCM [23]. Rawlins et al. [24] and Sheikh et al. [21] studied African American adolescent athletes: left ventricular hypertrophy was present in 5–7% of them (IVS > 12 mm, some of them up to 15 mm), compared to 0,6% of Caucasian athletes, while no African American sedentary adolescent showed this pattern. This result would suggest an exercise-correlated etiology of ventricular hypertrophy rather than ethnicity, and the possibility of an early cardiovascular remodeling. Recently no study in literature has followed time-correlated trends of left ventricular remodeling in adolescent athletes. Our study is unique from this point of view, showing that the different left ventricular remodeling between AA and CA, which is already present in pre-pubertal age, is maintained with development.

The IVS wall thickness is different in the two study groups, despite LVMI and RWT being similar. Firstly this aspect is difficult to interpret. It could be reasonable to think that the absence of change of the LVMI and RWT parameters could be related principally to the different LV diameters found.

The mild increase of LVMI in AA at 4-year evaluation could be interpreted as a consequence of training, being part of the athlete’s heart manifestation. This is suggested by normal morphological and functional parameters with the absence of contractile impairment.

Nowadays, determining ethnic-specific morphological and functional characteristics of left ventricular remodeling in elite athletes is a necessity. Since sports teams are multicultural, accurately recognizing ethnic-specific characteristics is an urgent need. Identifying specific echocardiographic criteria will help the Sports Physician to differentiate between physiological and pathological conditions, reducing the so-called “grey-zone”.

GLS has not been specifically determined in African American athletes yet. This is the reason why a comparison with other data is not possible. The results of our study show no difference and normal range values of GLS in athletes of the two ethnicities. Russo et al. [25] compared GLS between sedentary individuals of different descent, and lower values of GLS were found in African Americans (− 16,5 ± 3,5%) compared to Caucasians (− 17,5 ± 3,0%), despite similar values of ejection fraction. If these data and the results of our study were confirmed, a possible protective role of physical exercise in individuals of African American descent would be suggested.

### Peculiarities of our study

#### Some particular characteristics are present in our study.

Firstly, all the athletes belonged to the same team, trained with the same load and frequency and had the same lifestyle. Ethnicity was the only differential element among the athletes who were matched for sex, mean age, anthropometrical and clinical characteristics, deleting possible differences in results related to these determinants. This made it possible to evaluate specifically the contribution of ethnicity to left ventricular remodelling in elite athletes.

A second particular characteristic was the evaluation of the role of soccer in determining these cardiac adaptations, differently to other studies which were not sport-specific.

The mean age of the athletes enrolled was notable: they were evaluated before and during puberty, at ages that are well associated with important structural modifications. This allowed us to demonstrate that exercise-correlated left ventricular remodelling is already present early in life and that it is more marked in individuals of African American descent compared to Caucasians.

Our study was also structured so that left ventricular remodelling could be followed for over.

Four years and not only once, with the aim to see if the differences possibly found would be maintained or change during this period. Results show they are present after 4 years and slightly increase.

Lastly, GLS was specifically calculated in African American adolescent athletes compared to Caucasians for the first time.

### Limits of the study

This study is a particular investigation of myocardial function evaluated by ethnic characteristics. Some limits are evident and among these the small sample investigated represents the principal aspect in the results obtained. This appears to be particularly important in terms of the interpretation of the results of myocardial size and thickness.

In addition, many of the subjects enrolled were not followed for long because of normal seasonal team recruitment. Further studies will be necessary to confirm the present data.

Considering the aim of the study was to evaluate a homogeneous group of subjects, excluding as many confusing factors as possible, i.e. anthropometrics, lifestyle and load differences, the number of subjects that could be enrolled was consequently low.

## Data Availability

The data sets used and analyzed during the study are available from the corresponding author on request.
